# Association between Sleep Duration and Grip Strength in U.S. Older Adults: An NHANES Analysis (2011–2014)

**DOI:** 10.3390/ijerph20043416

**Published:** 2023-02-15

**Authors:** Jin Liu, Tianhao Zhang, Jia Luo, Shumin Chen, Dongfeng Zhang

**Affiliations:** Department of Epidemiology and Health Statistics, Qingdao University Medical College, Qingdao 266071, China

**Keywords:** sleep duration, grip strength, National Health and Nutrition Examination Survey, dose-response

## Abstract

Handgrip strength has been shown an indispensable biomarker for older adults. Furthermore, the association between sleep duration and grip strength in special populations (e.g., type 2 diabetics) has been previously documented. However, the association between sleep duration and grip strength has been less studied in older adults and the dose-response relationship is unclear. Therefore, we drew 1881 participants aged 60 years and older from the National Health and Nutrition Examination Survey (NHANES) 2011–2014 to explore their association and the dose-response relationship. Sleep duration was obtained through self-report. Grip strength data were obtained through a grip test using a handgrip dynamometer and divided into two categories: low grip strength and normal grip strength. Thus, dichotomized grip strength was used as a dependent variable. Poisson regression and restricted cubic spline were used for the main part of the analysis. We found that long sleep duration (≥9 h) was associated with a higher prevalence of low grip strength than the normal sleep duration (7–<9 h) group (IRR: 1.38, 95% CI: 1.12–1.69). Moreover, the gender-stratified analysis did not change the original results. This association was particularly pronounced and further strengthened among participants with normal weight (BMI < 25) (IRR: 2.30, 95% CI: 1.64–3.22) and participants aged 60–70 (IRR: 1.76, 95% CI: 1.40–2.22). In addition, with the increase in sleep duration, the multivariate-adjusted IRRs of low grip strength had a general downward trend at first, followed by a brief period of stability, and then presented an upward trend (*p*-value for non-linearity = 0.001). According to this study, we found that older adults who had long sleep duration had a higher risk of low grip strength. Muscle insulin utilization and muscle glucose metabolism are closely related to grip strength, so our research emphasizes the importance of maintaining normal sleep duration in older adults and suggests that older adults who sleep for a long period should pay more attention to their muscle health.

## 1. Introduction

Skeletal muscle strength and mass decrease more significantly in older adults [1]. Handgrip strength, an indispensable biomarker for older adults [2], is a reliable method of testing individuals’ total muscle strength and nutritional health [3]. Moreover, low grip strength is a well-known indicator of age-related physical decline [4]. Several previous studies demonstrated that low grip strength was associated with an increased risk of unfavorable health outcomes, such as depression [5], cognitive impairment [6], cardiovascular disease [7], and all-cause death [8]. Therefore, identifying factors that may contribute to the decline in grip strength with age in older adults is a public health priority. Muscle insulin utilization [9], muscle glucose metabolism [10], and growth hormone secretion [11] are closely related to grip strength. Unhealthy sleep duration may result in muscle insulin resistance and impaired muscle glucose metabolism by disturbing the circadian rhythm of skeletal muscle, resulting in decreased grip strength. In addition, previous epidemiological studies have indicated that more than half of older adults suffer from insomnia, frequent awakening, and sleep disruption [12]. Even in healthy people, disruption of sleep cycles can have serious effects on health and quality of life [13]. Therefore, it is necessary and meaningful to explore the relationship between sleep duration and grip strength.

Several researchers have explored this relationship, with inconsistent findings. A cross-sectional study of older males found that shorter sleep duration was associated with weaker grip strength [14]. A prospective study found that shorter total sleep duration was associated with decreased handgrip strength in older women over a 5-year period [15]. Another study of patients with type 2 diabetes found that long sleep duration was significantly associated with low grip strength [16]. In contrast, a previous study conducted in Taiwan indicated that sleep duration was positively associated with grip strength in the fully adjusted model [17]. Furthermore, Goldman SE noted no statistically significant association between total nightly sleep duration and grip strength in older females [18]. However, these studies had relatively small sample sizes and focused on older males, older females, or patients with type 2 diabetes. Moreover, the dose-response relationship between sleep duration and grip strength is unclear. Previous studies have found that age [19,20], gender [21,22], height, and weight [23] are significantly associated with grip strength. However, few studies have performed more meticulous stratified analyses according to age, gender, and BMI.

Using a sophisticated, large, layered, population-based sample collected from the “National Health and Nutrition Examination Survey (NHANES)”, we conducted cross-sectional research to: (1) explore the association between sleep duration and grip strength in older adults (2) explore the dose-response relationship between sleep duration and grip strength (3) examine the age-, gender-, and BMI-specific associations of sleep duration with grip strength. Our study visually demonstrates the dose-response relationship between sleep duration and grip strength, further underscoring the importance of maintaining normal sleep duration in older adults.

## 2. Materials and Methods

### 2.1. Data Source and Participants

The NHANES is a large, cross-sectional survey based on adults and children in the US on a two-year cycle. In addition, it is a complex multi-stage probability sample design that ensures a nationally representative sample of non-institutionalized US civilians. All participants were interviewed at home initially, then had their health checked in a mobile examination center [24]. The NCHS Ethics Review Board have given their approval to the research protocol, and each participant provided informed consent.

**Figure 1:**
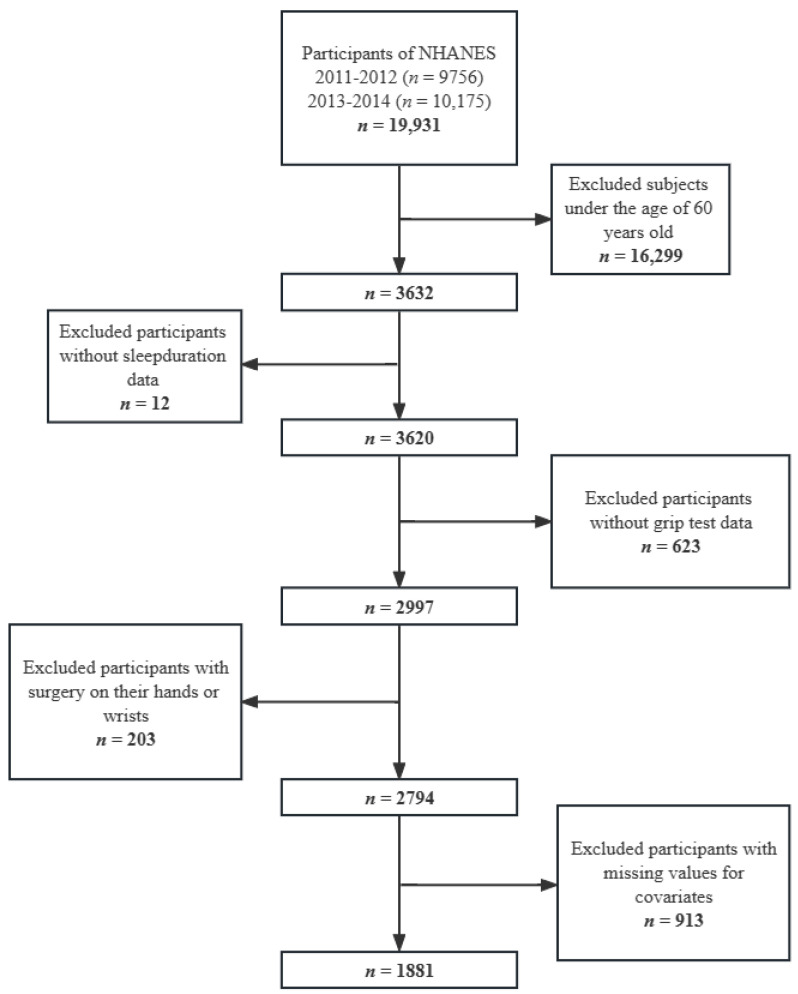
Flow chart of the screening process for the selection of eligible participants.

The data from 2 cycles of NHANES (2011–2012 and 2013–2014) were combined for the present analyses. During 2011–2014, 3632 participants aged 60 and older participated in the NHANES. We removed individuals with incomplete sleep duration questionnaires (*n* = 12) or with incomplete grip test data (*n* = 623) from the study. Individuals who had undergone surgery on the hand or wrist were further omitted (*n* = 203). In addition, we removed individuals with missing covariate data (*n* = 913). Finally, there were 1881 participants in this study who were 60 years of age or older (930 males and 951 females) (Figure 1).

### 2.2. Instruments

#### 2.2.1. Grip Strength

Grip strength data were obtained through a grip test using a handgrip dynamometer. Before the formal measurements were taken, a trained examiner talked each participant through the procedure and showed them how it was carried out. Participants first practiced based on the demonstration, and then, they were asked to squeeze the dynamometer as hard as they could. In addition, the participant’s dominant or non-dominant hand was randomly allocated to begin the test and each hand would be measured three times with an interval of 60 s. Each measurement was recorded in kilograms [25]. We used the reference equations for dominant hand grip strength proposed by Ying-Chih Wang et al. [26] (Male grip strength = −29.959 – 3.095 × 10^−5^ × (Age^3^) + 38.719 × (Height) + 0.113 × (Weight); Female grip strength = –22.717 – 1.920 × 10^−5^ × (Age^3^) + 30.360 × (Height) + 0.048 × (Weight)). At the same time, we brought each participant’s age, gender, height, and weight into the corresponding equations to obtain an individual-based reference value for dominant hand grip strength. Finally, the measured maximum grip strength of the dominant hand was compared with the reference value. Low grip strength was defined if the maximum grip strength of the dominant hand was less than the reference value. Finally, dichotomized grip strength (low grip strength or normal grip strength) was used as the dependent variable.

#### 2.2.2. Sleep Duration

The sleep investigations were performed by employing a computer-assisted personal interviewing system by qualified interviewers in the home. Sleep duration was estimated by self-report from one single question: “How much sleep do you usually get at night on weekdays or workdays?” and was further broken down into four categories: long (≥9 h), normal (7–<9 h), short (5–<7 h), and very short (<5 h), depending on the response to this question [27,28]. The distribution of sleep duration for enrolled participants is shown in Supplement Table S1.

#### 2.2.3. Covariates

A household-structured questionnaire was used to collect the following data: gender (male, female), age (60–70, ≥70 years), education level (below high school, high school, above high school), marital status (married/living with partner, widowed/divorced/separated/never married), race (Mexican American, other Hispanic, non-Hispanic White; non-Hispanic Black, other race), annual household income (<$20,000, ≥$20,000). BMI was determined as weight (kg) divided by height squared (kg/m^2^) and categorized into three groups (normal: <25 kg/m^2^, overweight: 25–<30 kg/m^2^, obese: ≥30 kg/m^2^). Consortium to Establish a Registry for Alzheimer’s Disease (CERAD) Word Learning sub-test, Animal Fluency test, and Digit Symbol Substitution Test (DSST) were used to assess cognitive function. We used the 25th percentile of the score as the cutoff point, which is consistent with the methods used in the published literature [29]. In addition, the score was categorized based on age to reduce the effect of age on cognitive function [30]. Disability was measured by two domains named functional limitations and activities of daily living (ADL) limitations [31]. Functional limitations were defined as self-reported “much difficulty” or “unable to do” one or more of six tasks (“walking 1/4 mile”, “walking up 10 steps”, “stooping/crouching/kneeling”, “standingup from an armless chair”, “lifting or carrying 10 lb”, and “walking between rooms on the same floor”). ADL limitations were defined as self-reported “much difficulty” or “unable to do” any of three tasks (“eat”, “dress”, and “get out of bed”). Work physical activity was divided into three groups (moderate, vigorous, other) as well as recreational physical activity. Smoking status was determined based on a question, “Have you smoked at least 100 cigarettes in your whole life?” (yes/no). Drinking status was determined by asking the question, “In any one year, have you had at least 12 drinks of any type of alcoholic beverage?” (yes/no). Participants were defined as hypertensive based on self-reported medical diagnosis, antihypertensive medicine usage, or a high blood pressure measurement value (systolic blood pressure 140 mm Hg and/or diastolic blood pressure 90 mm Hg). Participants were defined as diabetic based on self-reported medical diagnosis, insulin usage, or a blood glucose measurement value (fasting glucose ≥ 126 mg/dL and/or oral glucose tolerance test two hour glucose ≥ 200 mg/dL and/or glycohemoglobin ≥ 6.5%). Physical conditions were obtained through self-reported medical diagnosis: cancer (yes/no), stroke (yes/no), arthritis (yes/no), and coronary heart disease (yes/no). Patient health questionnaire-9 was used to assess depressive symptoms (score ≥ 10, score < 10). Total energy intake and caffeine intake were obtained from the 24-h dietary recall. The measurements of total testosterone in serum are performed using isotope dilution liquid chromatography tandem mass spectrometry (ID-LC-MS/MS). Since the variables of grip strength had been adjusted for age, sex, height, and weight, these four variables were not repeated for further analyses.

### 2.3. Statistical Analysis

First, we performed a descriptive statistical analysis based on the various categories of grip strength. Quantities (percentages) and medians (interquartile range) were utilized, respectively, to characterize qualitative and non-normally distributed data. Chi-square tests and Mann–Whitney U tests were conducted to compare fundamental information between the low grip strength group and the normal grip strength group. Next, we used participants with 7–<9 h sleep duration as reference. Louise-Anne McNutt et al. found that using logistic regression tends to overestimate the effect of exposure on outcomes in the presence of higher prevalence [32]. Jun Zhang et al. found that when the incidence of an outcome is common in the study population (>10%), the adjusted odds ratio derived from the logistic regression can no longer approximate the risk ratio [33]. In this study, the prevalence of low grip strength was high (34.02%). Therefore, Poisson regression analyses were carried out in order to evaluate the relationships between sleep duration and low grip strength. In addition, the results of incidence-rate ratios (IRRs) and 95% confidence intervals (CIs) calculations can be acquired simultaneously. Model 1 was a crude model. Model 2 adjusted for all covariates. Given differences in grip strength between gender, age groups, and BMI groups [26], we performed the stratified analyses by gender, age groups, and BMI groups. In addition, the dose-response relationship was evaluated by a restricted cubic spline with three knots (the 25th, 50th, and 75th percentiles of sleep duration) in the fully adjusted model. We further carried out a series of sensitivity analyses using the Poisson regression and logistic regression to test the robustness of the results. Sensitivity analysis 1 was performed by using the criteria of EWGSOP2 of grip strength (male = 27 kg, and female = 16 kg) [34]. Low grip strength was defined if the maximum grip strength was less than the reference value. Sensitivity analysis 2 was performed by excluding the participants with cognitive impairment. We weighted the analyses using NHANES recommendations to explicate the complexity of the sampling design [35]. Stata 15.0 (Stata Corporation, College Station, TX, USA) was utilized to perform all statistical analyses. Statistical significance was defined as a two-sided *p*-value less than 0.05.

## 3. Results

The characteristics of the participants in this study across grip strength are displayed in Table 1. Of the 1881 participants, females accounted for 50.56%, and the prevalence of low grip strength was 34.02%. Compared with participants with normal grip strength, participants with low grip strength tended to be obese, and more likely to have depressive symptoms, functional limitations, diabetes, stroke, arthritis, lower work activity, and lower total energy intake. Furthermore, participants with low grip strength had a longer sleep duration.

Table 2 displays the weighted IRRs with 95% CIs for low grip strength according to sleep duration. In model 1, long sleep duration was positively associated with low grip strength. In model 2, the positive association between long sleep duration and low grip strength was still significant, compared with normal sleep duration, the IRR (95% CI) of low grip strength for long sleep duration was 1.38 (1.12–1.69). On the contrary, the association between two categories of short sleep duration and low grip strength was consistently not statistically significant.

The associations of sleep duration with low grip strength stratified by gender, age, and BMI are shown in Table 3. The gender-based analysis did not alter the results that were statistically significant before stratification. The corresponding IRRs of the male group and the female group were 1.40 (1.04–1.89) and 1.35 (1.02–1.79). In age-stratified analyses, the association between long sleep duration and low grip strength was not statistically significant in the ≥70 age group. However, in the 60–70 age group, long sleep duration was still positively associated with low grip strength (IRR: 1.76, 95% CI: 1.40–2.22). In the stratified analyses by BMI, for participants with normal weight (BMI < 25), long sleep duration was also positively related to low grip strength risk (IRR: 2.30, 95% CI: 1.64–3.22). In the fully adjusted model, this association was not statistically significant in the overweight (BMI = 25–30) and obese (BMI ≥ 30) groups.

**Figure 2:**
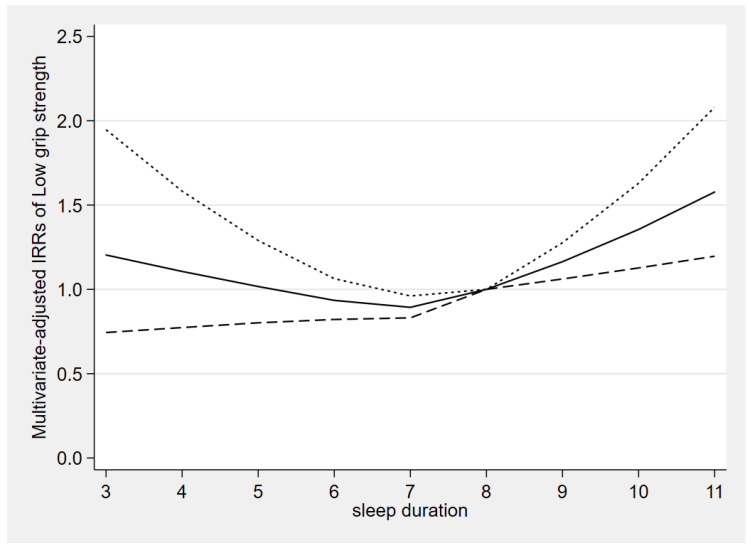
The dose-response relationship between sleep duration and the risk of low grip strength. Adjusted for race, marital status, education level, annual household income, physical activity, cognitive function, functional limitations, ADL limitations, caffeine intake, total energy intake, total testosterone level, smoking status, drinking status, medical history (hypertension, diabetes, cancer, stroke, arthritis, and coronary heart disease), as well as depressive symptoms. Reference is 8 h/day. The solid line and dash line represent the estimated IRRs and their 95% confidence intervals, respectively. IRR: incidence-rate ratios.

The dose-response relationship between sleep duration and low grip strength is presented in Figure 2. In older adults, with the increase in sleep duration, the multivariate-adjusted IRRs of low grip strength had a general downward trend at first, followed by a brief period of stability, and then presented an upward trend. Notably, the association became statistically significant when the sleep duration exceeded 8 h (Figure 2). In general, we found that the relationship between sleep duration and low grip strength was non-linear (*p*-value for non-linearity = 0.001).

In sensitivity analysis, the results of the relationship between sleep duration and low grip strength were not materially changed, which indicated the robustness of our findings (Supplement Tables S2 and S3).

## 4. Discussion

In this study, we analyzed data from the NHANES (2011–2014) database, which included 1881 participants aged at least 60 years old. Our research found that long sleep duration was associated with a higher risk of low grip strength. In addition, the gender-based analysis did not alter the results that were statistically significant before stratification. In the age-stratified analyses, long sleep duration was associated with a higher risk of low grip strength in the 60–70 age group. Furthermore, in the stratified analyses by BMI, we observed that for participants with normal weight (BMI < 25), long sleep duration was also positively related to low grip strength risk. Finally, in older adults, there was a non-linear relationship between sleep duration and low grip strength (*p*-value for non-linearity = 0.001).

Multiple epidemiological studies have examined the relationship between sleep and grip strength. Some studies found that long sleep duration was significantly associated with low grip strength [16,36,37,38,39]. In addition, these studies were consistent with our findings. Furthermore, a study of older Americans reported that insomnia symptoms were not associated with grip strength, which was similar to our result regarding no association between short sleep duration and low grip strength [40]. A study including 1274 men older than 65 years of age at baseline reported that testosterone had an inverted U-shaped relationship with sleep duration [41]. At the same time, the study also found a similar relationship between sleep duration and muscle mass and function [41]. Coincidentally, another cross-sectional study of 2862 men found an inverted U-shaped relationship between total nightly sleep duration and grip strength, and this relationship persisted after adjusting for daytime sleep [14]. However, a previous study conducted in Taiwan indicated that sleep duration was positively associated with grip strength in the fully adjusted model [17]. It is noteworthy that the small sample size can influence the results. Goldman SE noted no statistically significant association between total nightly sleep duration and grip strength in older females [18]. Another prospective study found that shorter total sleep duration was associated with decreased handgrip strength in older women over a 5-year period [15]. The different findings may have resulted from varying study subjects, age ranges, and confounding variables.

The underlying mechanisms between sleep duration and grip strength are not fully established, and further research is needed to investigate the related biological mechanisms. Both studies by Auyeung and Chen demonstrated that extreme sleep duration, independent of muscle mass, may have an impact on muscular performance [39,41]. There are various possible explanations for why excessive sleep duration might lead to low grip strength. Firstly, the circadian clock plays a critical role in skeletal muscle physiology. Not only does it control the development, repair, and maintenance of muscle mass, but it also controls the structure and function of skeletal muscle [42]. Above all, it is also involved in muscle metabolism [42]. Unhealthy sleep duration as well as sleep disorders may lead to muscle insulin resistance and altered muscle glucose metabolism by disturbing the circadian rhythm of skeletal muscle, resulting in decreased muscle function and grip strength [9]. Second, a previous study has found that sleep duration was associated with the production of growth hormone, insulin-like growth factor 1, and testosterone, which in turn might affect muscle function [11]. In addition, both ends of sleep duration may have different pathomechanisms [39]. Finally, a previous genome-wide analysis of sleep duration indicated that neuropsychiatric and metabolic diseases shared some of the same genetic pathways as sleep traits [43]. This suggests that analysis from a genetic perspective may assist in explaining this finding.

This study has several advantages. First, our study was based on high-quality and nationally representative data from the NHANES. Second, we used a more robust Poisson regression to explore the relationship between sleep duration and low grip strength. Third, dose-response relationships between sleep duration and low grip strength were appraised in this study. Finally, gender, age, and BMI differences were also carefully assessed. However, there were a few limitations to consider. First of all, the cross-sectional design limited further causal inferences. In addition, our result cannot directly support the possible underlying mechanisms of the association between hand grip strength and sleep duration. In addition, sleep duration was obtained by self-report, hence this study might be subject to recall bias. However, objective methods, such as polysomnography, to accurately measure the sleep duration is usually not feasible in large-scale surveys. Using estimated sleep duration instead of actual sleep duration tends to weaken the association of sleep duration with outcome events, so the true effect of sleep duration on grip strength may be more significant than what we have observed in our study. The previous study [44] have shown subjective estimates of sleep duration and more precise measurements are highly correlated. Third, although we included many covariates in the fully adjusted model, there was still the possibility of other unmeasured confounders due to data limitations. Finally, we cannot perform further subgroup analyses of age and BMI in different genders by virtue of the limited sample size.

## 5. Conclusions

In conclusion, we found that long sleep duration was associated with a higher risk of low grip strength. In the sex-stratified analyses, we did not find sex differences in this association. In the age-stratified analyses, long sleep duration was associated with a higher risk of low grip strength in the 60–70 age group. Furthermore, in the stratified analyses by BMI, we observed that for participants with normal weight (BMI < 25), long sleep duration was also positively related to low grip strength risk. Furthermore, there was a nonlinear dose-response relationship between sleep duration and low grip strength. Our research may serve as a framework to better understand the importance of normal sleep duration in the prevention of low grip strength in older adults.

## Figures and Tables

**Table 1:** Baseline characteristics of participants, NHANES 2011–2014 (*n* = 1881).

	Low Grip Strength	Normal Grip Strength	*p* Value
Number of participants (%) ^a^	640 (34.02)	1241 (65.98)	
Age, *n* (%) ^a^			0.996
60–70 years	353 (55.16)	687 (55.36)	
≥70 years	287 (44.84)	554 (44.64)	
Gender, *n* (%) ^a^			0.642
Male	310 (48.44)	620 (49.96)	
Female	330 (51.56)	621 (50.04)	
Body mass index, *n* (%) ^a^			<0.05
<25 kg/m^2^	172 (26.88)	343 (27.64)	
25 to <30 kg/m^2^	196 (30.63)	464 (37.39)	
≥30 kg/m^2^	272 (42.50)	434 (34.97)	
Race, *n* (%) ^a^			0.226
Mexican American	56 (8.75)	97 (7.82)	
Other Hispanic	56 (8.75)	120 (9.67)	
Non-Hispanic White	369 (57.66)	620 (49.96)	
Non-Hispanic Black	120 (18.75)	298 (24.01)	
Other races	39 (6.09)	106 (8.54)	
Marital status, *n* (%) ^a^			0.848
Living with partner/married	376 (58.75)	754 (60.76)	
Widowed/separated/divorced/ never married	264 (41.25)	487 (39.24)	
Educational level, *n* (%) ^a^			0.919
<high school	132 (20.63)	271 (21.84)	
High school	154 (24.06)	306 (24.66)	
>high school	354 (55.31)	664 (53.51)	
Annual household income, *n* (%) ^a^			0.175
<$20,000	157 (24.53)	270 (21.76)	
≥$20,000	483 (75.47)	971 (78.24)	
Work activity, *n* (%) ^a^			<0.01
Vigorous	50 (7.81)	166 (13.38)	
Moderate	131 (20.47)	269 (21.68)	
Other	459 (71.72)	806 (64.95)	
Recreational activity, *n* (%) ^a^			0.304
Vigorous	54 (8.44)	144 (11.60)	
Moderate	211 (32.97)	440 (35.46)	
Other	375 (58.59)	657 (52.94)	
Sleep duration, *n* (%) ^a^			<0.05
<5 h	24 (3.75)	53 (4.27)	
5 to <7 h	176 (27.50)	364 (29.33)	
7 to <9 h	357 (55.78)	716 (57.70)	
≥9 h	83 (12.97)	108 (8.70)	
Low CERAD Test Performance, *n* (%) ^a^	163 (25.47)	267 (21.51)	0.153
Low Animal Fluency Test Performance, *n* (%) ^a^	168 (26.25)	315 (25.38)	0.140
Low Digit Symbol Test Performance, *n* (%) ^a^	154 (24.06)	266 (21.43)	0.061
Cognitive impairment, *n* (%) ^a^	294 (45.94)	537 (43.27)	0.102
Functional limitations, *n* (%) ^a^	223 (34.84)	226 (18.21)	<0.01
ADL limitations, *n* (%) ^a^	20 (3.13)	26 (2.10)	0.685
Smoke at least 100 cigarettes in life, *n* (%) ^a^	315 (49.22)	613 (49.40)	0.394
Had at least 12 alcohol drink a year, *n* (%) ^a^	450 (70.31)	876 (70.59)	0.531
Hypertension, *n* (%) ^a^	514 (80.31)	970 (78.16)	0.362
Diabetes, *n* (%) ^a^	229 (35.78)	337 (27.16)	<0.01
Ever told you had a cancer, *n* (%) ^a^	136 (21.25)	249 (20.06)	0.931
Ever told you had a stroke, *n* (%) ^a^	55 (8.59)	64 (5.16)	<0.05
Ever told you had arthritis, *n* (%) ^a^	363 (56.72)	530 (42.71)	<0.01
Ever told you had coronary heart disease, *n* (%) ^a^	68 (10.63)	107 (8.62)	0.158
Depressive symptoms, *n* (%) ^a^	65 (10.16)	81 (6.53)	<0.05
Caffeine intake (mg/d), median (IQR) ^b^	109.75 (164.50)	108.00 (177.50)	0.676
Total energy intake (kcal/d), median (IQR) ^b^	1715.25 (866.5)	1765.00 (811.50)	<0.05
Total testosterone level (ng/dL), median (IQR) ^b^	44.64 (327.65)	61.92 (355.01)	0.213

Data are the number of participants (weighted percentage) or medians (interquartile ranges). ^a^ Chi-square test was used to compare the percentage between participants with and without low grip strength. ^b^ Mann-Whitney U test was used to compare the mean values between participants with and without low grip strength.

**Table 2:** Weighted IRRs and 95% CIs of low grip strength according to sleep duration, NHANES 2011–2014.

Sleep Duration (h/Day)	Cases/Participants (Prevalence Values)	Model 1 ^a^	Model 2 ^b^
		IRR (95% CI)	IRR (95% CI)
<5	24/77 (31.17%)	1.10 (0.68–1.75)	1.08 (0.68–1.72)
5–<7	176/540 (32.59%)	1.07 (0.81–1.41)	1.03 (0.78–1.39)
7–<9	357/1073 (33.27%)	1.00 (reference)	1.00 (reference)
≥9	83/191 (43.46%)	1.52 (1.22–1.90) **	1.38 (1.12–1.69) **

Calculated using Poisson regression. IRR, incidence-rate ratio; CI, confidence interval. ^a^ Model 1 is the unadjusted model. ^b^ Model 2 is adjusted for race, marital status, education level, annual household income, physical activity, cognitive function, functional limitations, ADL limitations, caffeine intake, total energy intake, total testosterone level, smoking status, drinking status, medical history (hypertension, diabetes, cancer, stroke, arthritis, and coronary heart disease), as well as depressive symptoms. ** *p* < 0.01.

**Table 3:** Weighted IRRs and 95% CIs of low grip strength according to sleep duration stratified by gender age and BMI, NHANES 2011–2014.

Sleep Duration (h/Day)	Cases/Participants (Prevalence Values)	Model 1 ^a^	Model 2 ^b^
		IRR (95% CI)	IRR (95% CI)
		Males	
<5	8/29 (27.59%)	1.28 (0.60–2.74)	1.50 (0.73–3.08)
5–<7	88/258 (34.11%)	1.17 (0.80–1.73)	1.13 (0.76–1.69)
7–<9	172/543 (31.68%)	1.00 (reference)	1.00 (reference)
≥9	42/100 (42.00%)	1.49 (1.05–2.11) **	1.40 (1.04–1.89) *
		Females	
<5	16/48 (33.33%)	0.97 (0.59–1.61)	0.93 (0.57–1.51)
5–<7	88/282 (31.21%)	0.98 (0.71–1.37)	0.94 (0.68–1.29)
7–<9	185/530 (34.91%)	1.00 (reference)	1.00 (reference)
≥9	41/91 (45.05%)	1.56 (1.18–2.06) **	1.35 (1.02–1.79) *
		60–70 years	
<5	18/53 (33.96%)	1.65 (0.68–3.99)	1.55 (0.95–2.51)
5–<7	103/325 (31.69%)	1.06 (0.59–1.90)	1.00 (0.70–1.44)
7–<9	197/586 (33.62%)	1.00 (reference)	1.00 (reference)
≥9	35/76 (46.05%)	3.19 (1.86–5.46) **	1.76 (1.40–2.22) **
		≥70 years	
<5	6/24 (25.00%)	0.65 (0.20–2.13)	0.71 (0.26–1.96)
5–<7	73/215 (33.95%)	1.17 (0.74–1.86)	1.04 (0.75–1.44)
7–<9	160/487 (32.85%)	1.00 (reference)	1.00 (reference)
≥9	48/115 (41.74%)	1.38 (0.80–2.38)	1.08 (0.79–1.48)
		<25 kg/m^2^	
<5	7/18 (38.89%)	1.45 (0.60–3.49)	1.44 (0.68–3.02)
5–<7	46/142 (32.39%)	1.11 (0.72–1.72)	1.12 (0.74–1.69)
7–<9	92/309 (29.77%)	1.00 (reference)	1.00 (reference)
≥9	27/46 (58.70%)	2.32 (1.67–3.22) **	2.30 (1.64–3.22) **
		25–30 kg/m^2^	
<5	6/17 (35.29%)	1.03 (0.34–3.17)	1.19 (0.34–4.24)
5–<7	43/173 (24.86%)	0.93 (0.53–1.63)	0.91 (0.50–1.66)
7–<9	122/397 (30.73%)	1.00 (reference)	1.00 (reference)
≥9	25/73 (34.25%)	1.38 (0.91–2.10)	1.32 (0.87–2.01)
		≥30 kg/m^2^	
<5	11/42 (26.19%)	0.88 (0.46–1.67)	0.80 (0.45–1.44)
5–<7	87/225 (38.67%)	1.04 (0.77–1.40)	1.04(0.78–1.37)
7–<9	143/367 (38.96%)	1.00 (reference)	1.00 (reference)
≥9	31/72 (43.06%)	1.23 (0.95–1.58)	1.03 (0.84–1.27)

Calculated using Poisson regression. IRR, incidence-rate ratio; CI, confidence interval. ^a^ Model 1 is the unadjusted model. ^b^ Model 2 is adjusted for race, marital status, education level, annual household income, physical activity, cognitive function, functional limitations, ADL limitations, caffeine intake, total energy intake, total testosterone level, smoking status, drinking status, medical history (hypertension, diabetes, cancer, stroke, arthritis, and coronary heart disease), as well as depressive symptoms. * *p* < 0.05, ** *p* < 0.01.

## Data Availability

The datasets supporting the conclusions of this article are publicly available from the NHANES (https://www.cdc.gov/nchs/nhanes/index.htm). Accessed on 10 August 2022.

## Supplementary Materials

The following supporting information can be downloaded at: https://www.mdpi.com/article/10.3390/ijerph20043416/s1, Table S1: Baseline sleep duration characteristics of participants, NHANES 2011–2014 (*n* = 1881); Table S2: Weighted ORs and 95% CIs of low grip strength according to sleep duration, NHANES 2011–2014; Table S3: Weighted IRRs and 95% CIs of low grip strength according to sleep duration, NHANES 2011–2014.
