# Supplementary material for: Association between Sleep Duration and Grip Strength in U.S. Older Adults: An NHANES Analysis (2011–2014)
Source: Int J Environ Res Public Health. 2023 Feb 15;20(4):3416. doi: 10.3390/ijerph20043416 (PMC9964571; doi:10.3390/ijerph20043416)
Supplement: Supplementary file 1 [file ijerph-20-03416-s001.zip › ijerph-2085692-supplementary.pdf]

Table S1. Baseline sleep duration characteristics of participants, NHANES 2011–2014 (N = 1881).

	Number of participants	Percent
Sleep duration		
<5 h	77	4.09%
5 to <7 h	540	28.71%
7 to <9 h	1073	57.04%
≥9 h	191	10.15%

Table S2. Weighted ORs and 95% CIs of low grip strength according to sleep duration, NHANES 2011-2014.

Sleep duration (h/day)	Cases/Participants (Prevalence values)	Model 1 [a]	Model 2 [b]
		OR (95% CI)	OR (95% CI)
**<5**	2/77 (2.60%)	1.97 (0.38–10.30)	2.72 (0.23–32.19)
**5-<7**	18/540 (3.33%)	1.13 (0.59–2.17)	1.14 (0.50–2.59)
**7-<9**	38/1073 (3.54%)	1.00 (reference)	1.00 (reference)
**≥9**	20/191 (10.47%)	4.27 (2.51–7.28) **	3.28 (1.65–6.51) **

Calculated using binary logistic regression. OR, odds ratio; CI, confidence interval.

[a] Model 1 is the unadjusted model.

[b] Model 2 is adjusted for age, BMI, race, marital status, education level, annual household income, physical activity, cognitive function, functional limitations, ADL limitations, caffeine intake, total energy intake, total testosterone level, smoking status, drinking status, medical history (hypertension, diabetes, cancer, stroke, arthritis and coronary heart disease), as well as depressive symptoms.

**$p < 0.01$

Table S3. Weighted IRRs and 95% CIs of low grip strength according to sleep duration, NHANES 2011-2014.

Sleep duration (h/day)	Cases/Participants (Prevalence values)	Model 1 [a]	Model 2 [b]
		IRR (95% CI)	IRR (95% CI)
**<5**	9/34 (26.47%)	0.92 (0.41–2.09)	0.86 (0.38–1.92)
**5-<7**	95/304 (31.25%)	1.04 (0.74–1.46)	1.02 (0.70–1.46)
**7-<9**	202/616 (32.79%)	1.00 (reference)	1.00 (reference)
**≥9**	40/96 (41.67%)	1.47 (1.17–1.85) **	1.36 (1.06–1.75) *

Calculated using Poisson regression. IRR, incidence-rate ratio; CI, confidence interval.

[a] Model 1 is the unadjusted model.

[b] Model 2 is adjusted for race, marital status, education level, annual household income, physical activity, cognitive function, functional limitations, ADL limitations, caffeine intake, total energy intake, total testosterone level, smoking status, drinking status, medical history (hypertension, diabetes, cancer, stroke, arthritis and coronary heart disease), as well as depressive symptoms.

*$p < 0.05$

**$p < 0.01$
